# Crystal structure, Hirshfeld surface analysis and DFT study of 6-bromo-3-(5-bromo­hex­yl)-2-[4-(di­methyl­amino)­phen­yl]-3*H*-imidazo[4,5-*b*]pyridine

**DOI:** 10.1107/S2056989020008889

**Published:** 2020-07-10

**Authors:** Zainab Jabri, Nada Kheira Sebbar, Tuncer Hökelek, Joel T. Mague, Safia Sabir, Youssef Kandri Rodi, Khalid Misbahi

**Affiliations:** a Laboratory of Applied Organic Chemistry, Sidi Mohamed Ben Abdellah University, Faculty of Sciences and Techniques, Road Immouzer, BP 2202 Fez, Morocco; b Laboratoire de Chimie Bioorganique Appliquée et Environnement Equipe de Chimie, Bioorganique Appliquée, Faculté des Sciences, Université Ibn Zohr, Agadir, Morocco; c Laboratoire de Chimie Organique Hétérocyclique URAC 21, Pôle de Compétence Pharmacochimie, Av. Ibn Battouta, BP 1014, Faculté des Sciences, Université Mohammed V, Rabat, Morocco; d Department of Physics, Hacettepe University, 06800 Beytepe, Ankara, Turkey; e Department of Chemistry, Tulane University, New Orleans, LA 70118, USA

**Keywords:** crystal structure, C—H⋯π(ring) inter­action, imidazo­pyridine, DFT calculation, Hirshfeld surface analysis

## Abstract

In the title mol­ecule, C_20_H_24_Br_2_N_4_, the imidazo­pyridine moiety is not planar as indicated by the dihedral angle of 2.0 (2)° between the constituent rings; the 4-di­methyl­amino­phenyl ring is inclined to the mean plane of the imidazole ring by 27.4 (1)°. In the crystal, two sets of C—H⋯π(ring) inter­actions form stacks of mol­ecules extending parallel to the *a-*axis direction. Hirshfeld surface analysis indicates that the most important contributions to the crystal packing are from H⋯H (42.2%), H⋯C/C⋯H (23.1%) and H⋯Br/Br⋯H (22.3%) inter­actions. The optimized structure calculated using density functional theory (DFT) at the B3LYP/ 6–311 G(d,p) level is compared with the experimentally determined structure in the solid state. The calculated HOMO–LUMO energy gap is 2.3591 eV.

## Abstract 2

The 5-bromo­pentyl chain is oriented so that the bromine atom is *ca* 4.4 Å from one of the methyl C atoms of the di­methyl­amino group. In the crystal, two sets of inversion-related C—H⋯π(ring) inter­actions form stacks of mol­ecules extending along the *a*-axis direction.

## Chemical context   

The family of nitro­genous drugs, particularly those containing the imidazo­pyridine moiety, is important in medicinal chemistry because of their wide range of pharmacological activities such as anti­cancer, anti-inflammatory, anti­bacterial, anti-tuberculosis, anti-glycation anti-analgesic and anti­fungal properties, and their anti­oxidant potential. In particular, imadazo[4,5-*b*]pyridine derivatives inhibit the P-glycoprotein, which could reverse the multidrug resistance of cancer cells (Bourichi *et al.*, 2018 ▸). They are also inhibitors of type 2 diabetes because of their ability to inhibit the Baker’s yeast α-glucosidase enzyme, and are inhibitors of one or more proteins in the treatment of disorders characterized by the activation of Wnt pathway signalling (for example: cancer, abnormal cellular proliferation, angiogenesis, fibrotic disorders, bone or cartilage diseases and osteoarthritis), and of genetic and neurological diseases such as PAK4 kinase 4 inhibitor activated by p21 and aurora kinase inhibitors. Imadazo[4,5-*b*]pyridine derivatives are also therapeutic agents for dysferlinopathies through phenotypic screening on patient-induced pluripotent stem cells (Takada *et al.*, 2019 ▸).

Given the wide range of theraputic applications for such compounds, we have already reported a route for the preparation of imidazo[4,5-*b*]pyridine derivatives using *N*-alkyl­ation reactions carried out with di-halogenated carbon chains (Jabri *et al.*, 2020 ▸); a similar approach yielded the title compound, C_20_H_24_Br_2_N_4_,(I). Besides the synthesis, we also report the mol­ecular and crystal structures along with a Hirshfeld surface analysis and a density functional theory (DFT) computational calculation carried out at the B3LYP/6–311 G(d,p) level.
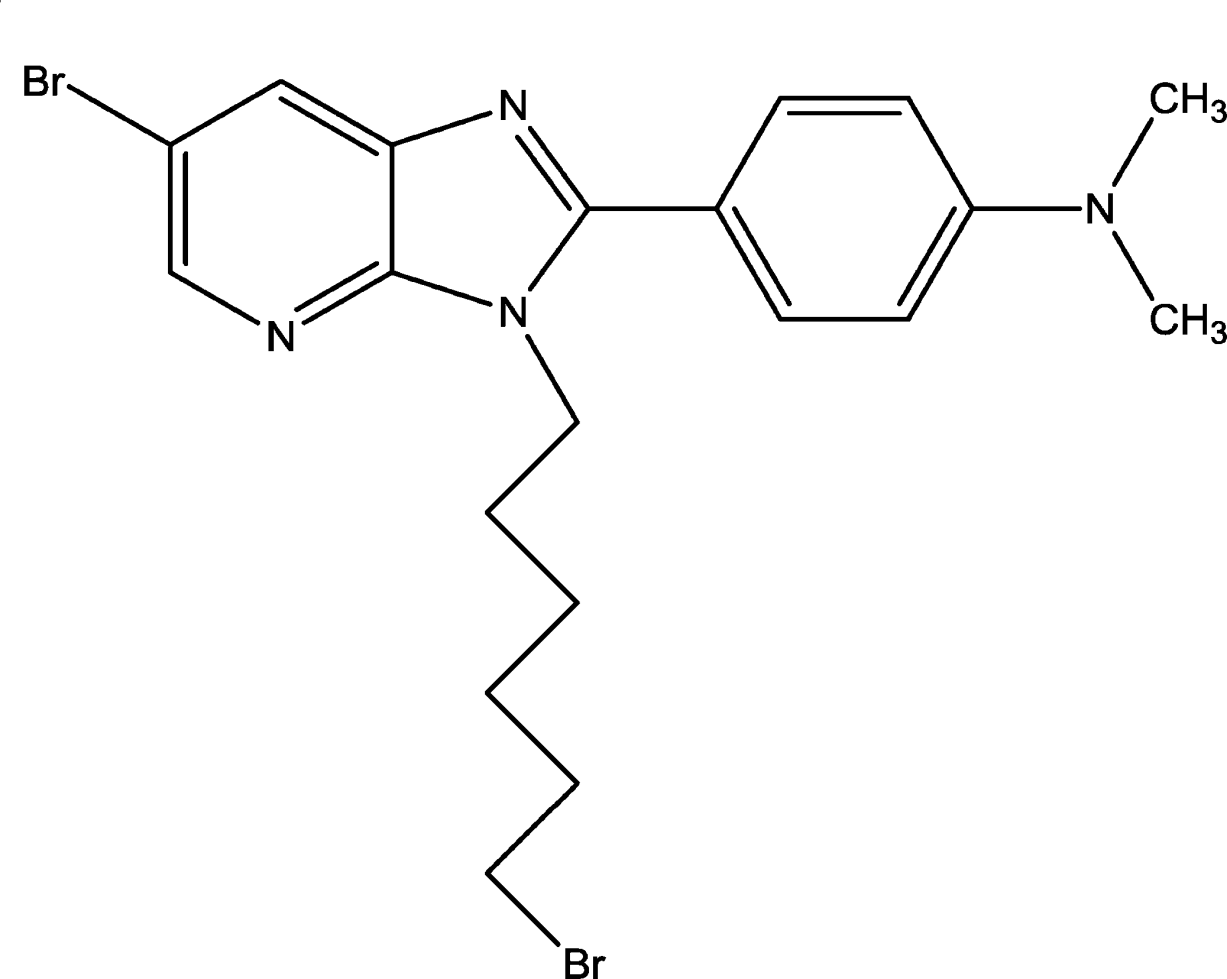


## Structural commentary   

**Figure 1:**
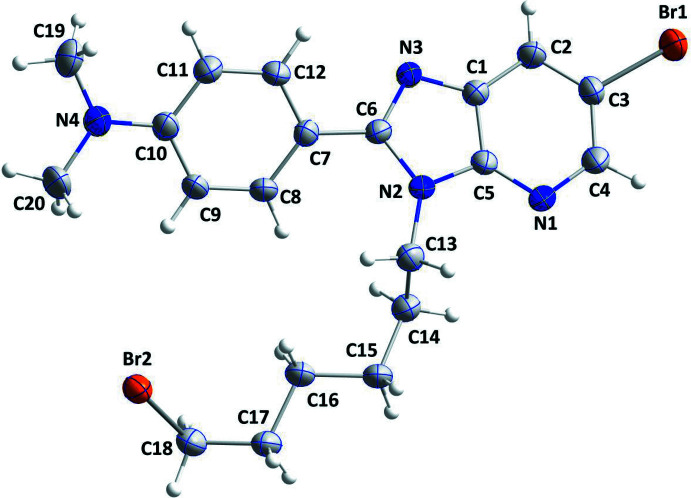
The asymmetric unit of the title compound with the atom-numbering scheme. Displacement ellipsoids are drawn at the 50% probability level.

The mol­ecular structure of (I) is depicted in Fig. 1 ▸. The imidazo­pyridine moiety is not planar, as indicated by the dihedral angle of 2.0 (3)° between the constituent rings. The ring of the 4-di­methyl­amino­phenyl moiety is inclined to the mean plane of the imidazole ring by 27.4 (1)°. The 5-bromo­pentyl chain is oriented in an arc-like form around the periphery of the 4-di­methyl­amino­phenyl moiety so that the terminal Br2 atom of the chain is only 4.36 (6) Å from one of the methyl C atoms (C20; Fig. 1 ▸).

## Supra­molecular features   

**Figure 2:**
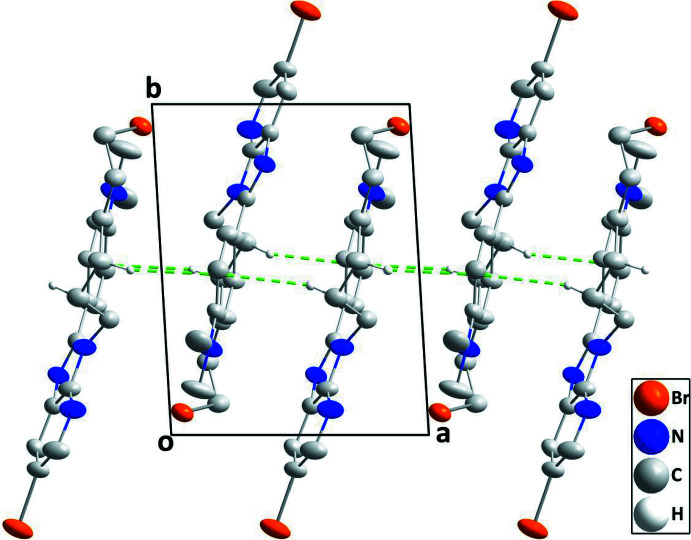
A portion of one stack of mol­ecules viewed along the *c-*axis direction with the C—H⋯π(ring) inter­actions depicted by green dashed lines.

In the crystal, stacks of mol­ecules extending along the *a-*axis direction are formed by inversion-related C14—H14*B*⋯*Cg*3^i^ and C15—H15*A*⋯*Cg*3^ii^ inter­actions [symmetry codes: (i) 1 − *x*, 1 − *y*, 1 − *z*; (ii) 2 − *x*, 1 − *y*, 1 − *z*] where *Cg*3 is the centroid of the C7–C12 phenyl ring (Fig. 2 ▸, Table 1 ▸).

## Hirshfeld surface analysis   

**Figure 3:**
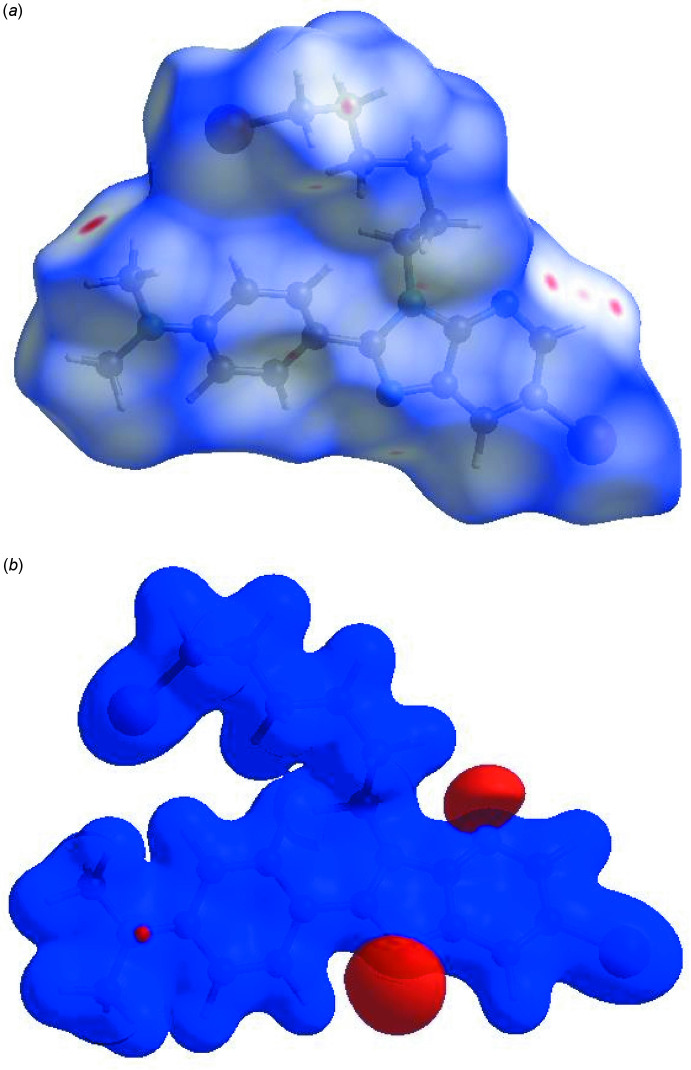
(*a*) View of the three-dimensional Hirshfeld surface of the title compound, plotted over *d*_norm_ in the range of −0.0709 to 1.1781 a.u. (*b*) View of the three-dimensional Hirshfeld surface of the title compound plotted over electrostatic potential energy in the range −0.0500 to 0.0500 a.u. using the STO-3 G basis set at the Hartree–Fock level of theory.

**Figure 4:**
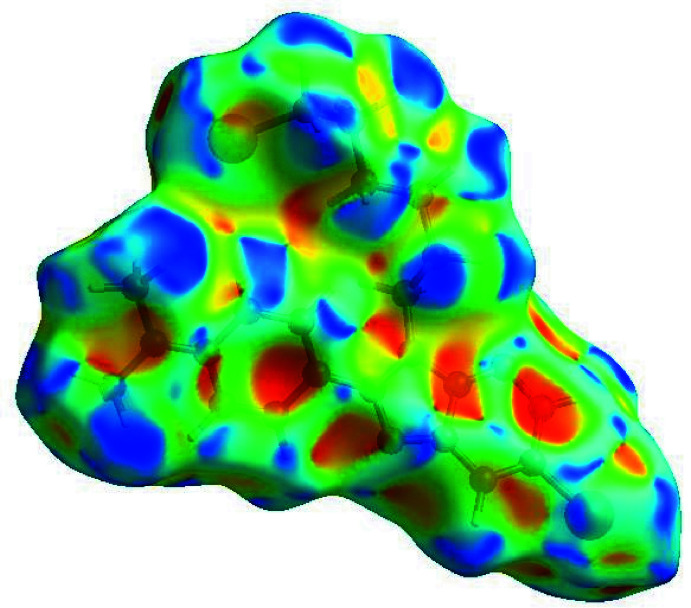
Hirshfeld surface of the title compound plotted over shape-index.

**Figure 5:**
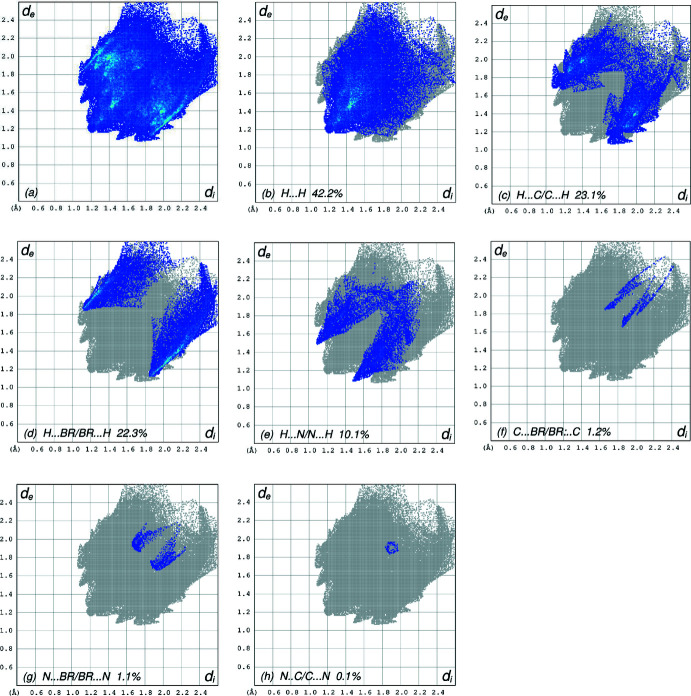
The full two-dimensional fingerprint plots for the title compound, showing (*a*) all inter­actions, and delineated into (*b*) H⋯H, (*c*) H⋯C/C⋯H, (*d*) H⋯Br/Br⋯H, (*e*) H⋯N/N⋯H, (*f*) C⋯Br/Br⋯C, (*g*) N⋯Br/Br⋯N and (*h*) N⋯C/C⋯N inter­actions. The *d*_i_ and *d*_e_ values are the closest inter­nal and external distances (in Å) from given points on the Hirshfeld surface.

In order to visualize the inter­molecular inter­actions in the crystal of the title compound, a Hirshfeld surface (HS) analysis (Hirshfeld, 1977 ▸) was carried out by using *Crystal Explorer 17.5* (Turner *et al.*, 2017 ▸). A view of the three-dimensional Hirshfeld surface of (I), plotted over *d*_norm_ and electrostatic potential are shown in Fig. 3 ▸ *a* and3*b*. The shape-index of the HS reveals that there are no significant π–π inter­actions in (I), as shown in Fig. 4 ▸. The overall two-dimensional fingerprint plot (McKinnon *et al.*, 2007 ▸) is shown in Fig. 5 ▸ *a*, while those delineated into H⋯H, H⋯C/C⋯H, H⋯Br/Br⋯H, H⋯N/N⋯H, C⋯Br/Br⋯C, N⋯Br/Br⋯N and N⋯C/C⋯N contacts are illustrated in Fig. 5 ▸ *b*–*h*, respectively, together with their relative contributions to the Hirshfeld surface. The most important inter­action is H⋯H, contributing 42.2% to the overall crystal packing, which is reflected in Fig. 5 ▸ *b* as widely scattered points of high density due to the large hydrogen content of the mol­ecule, with the tip at *d*_e_ = *d*_i_ = 1.18 Å. In the presence of C—H⋯π inter­actions, the pair of characteristic wings in the fingerprint plot delineated into H⋯C/C⋯H contacts (23.1% contribution to the HS), Fig. 5 ▸ *c*, has the tips at *d*_e_ + *d*_i_ = 2.76 Å. The pair of scattered points of spikes in the fingerprint plot delineated into H⋯Br/Br⋯H, Fig. 5 ▸ *d* (22.3%), have the tips at *d*_e_ + *d*_i_ = 2.95 Å. The H⋯N/N⋯H contacts, Fig. 5 ▸ *e* (10.1%), have the tips at *d*_e_ + *d*_i_ = 2.56 Å. The C⋯Br/Br⋯C contacts, Fig. 5 ▸ *f*, contribute 1.2% to the HS and appear as a pair of scattered points of spikes with the tips at *d*_e_ + *d*_i_ = 3.50 Å. The N⋯Br/Br⋯N contacts, Fig. 5 ▸ *g*, contribute 1.1% to the HS appearing as pair of scattered points of spikes with the tips at *d*_e_ + *d*_i_ = 3.59 Å. Finally, the N⋯C/C⋯N contacts, Fig. 5 ▸ *h*, make only 0.1% contribution to the HS and have a low-density distribution of points.

## DFT calculations   

**Figure 6:**
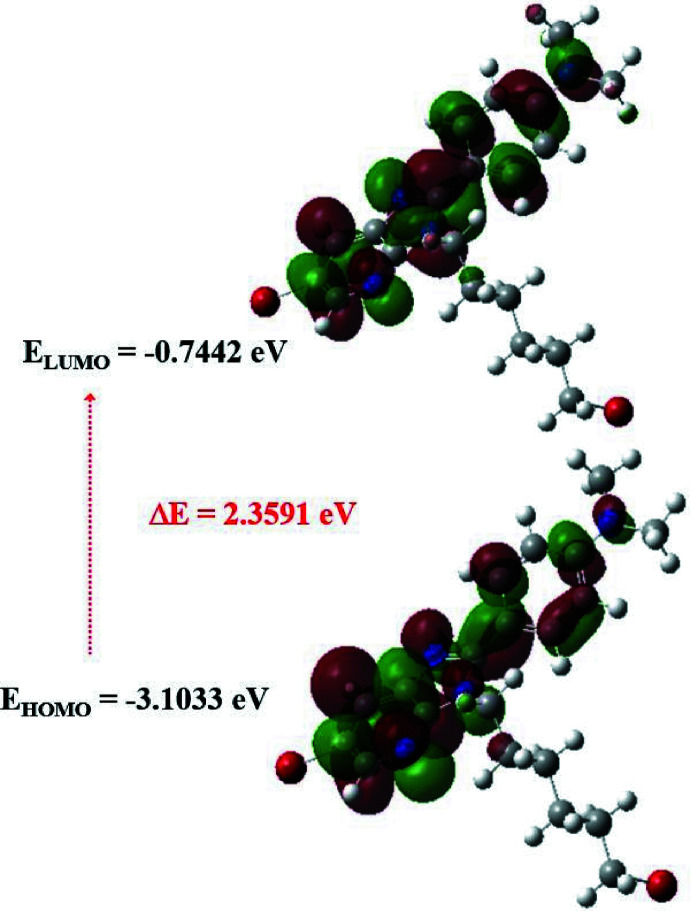
The energy band gap of (I).

The optimized structure of (I) in the gas phase was calculated by density functional theory (DFT) using a standard B3LYP functional and the 6–311 G(d,p) basis-set (Becke, 1993 ▸) as implemented in *GAUSSIAN 09* (Frisch *et al.*, 2009 ▸). The theoretical and experimental results related to bond lengths and angles are in good agreement (Table 2 ▸). Calculated numerical values for (I) including electronegativity (χ), hardness (η), potential (μ), electrophilicity (ω) and softness (*σ*) are collated in Table 3 ▸. The electron transition from the HOMO to the LUMO energy level is shown in Fig. 6 ▸. The HOMO and LUMO are localized in the plane extending ove the whole 6-bromo-3-(5-bromo­pent­yl)-2-[4-(di­methyl­amino)­phen­yl]-3-*H*-imidazo[4,5-*b*]pyridine system. The energy band gap [Δ*E* = *E*_LUMO_ − *E*_HOMO_] of the mol­ecule is 2.3591 eV, and the frontier mol­ecular orbital energies, *E*_HOMO_ and *E*_LUMO_, are −3.1033 and −0.7442 eV, respectively.

## Database survey   

**Figure 7:**
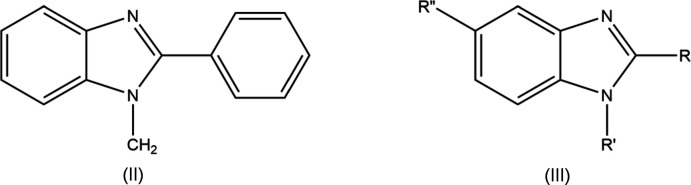
Structural fragments (II) and (III) used in the database search.

A search of the Cambridge Structural Database (CSD version 5.40, updated to March 2020; Groom *et al.*, 2016 ▸) with fragment (II) (Fig. 7 ▸) and excluding metal complexes gave seven matches. Of these, two had a –CH_2_CH_2_ *X*– (*X* = O, NH) chain connecting a saturated nitro­gen atom [corresponding to N2 in (I)] to an *ortho* position of the phenyl ring and so were considered less comparable to (I) than the remainder, which can be represented by the general structure (III) (Fig. 7 ▸). For *R* = Ph and *R*" = Br, examples are UCOXES (*R*′ = CH_2_COOC_2_H_5_; Hjouji *et al.*, 2016 ▸), UNUWIK [*R*′ = (1-benzyl-1*H*-1,2,3-triazol- 5-yl)methyl; Ouzidan *et al.*, 2011*a* ▸] and URAQOU [*R*′ = (2-oxooxazolidin-3-yl)ethyl; Ouzidan *et al.*, 2011*b* ▸]. For *R* = 4-ClC_6_H_4_ and *R*" = Br there are two reports of the compound with *R*′ = 1-octyl-1*H*-1,2,3-triazol-4-yl)methyl [XITLUK (Bourichi *et al.*, 2019*a* ▸) and XITLUK01 (Bourichi *et al.*, 2019*b* ▸)]. The dihedral angle between the plane of the 4-di­methyl­amino­phenyl group and the mean plane of the imidazo­pyridine unit is *ca* 19° in XITLUK and *ca* 49° in UCOXES. Of all of these related structures, (I) is the only one with the substituent on nitro­gen approximately coplanar with the imidazo­pyridine unit. In UCOXES, this substituent is directed outward and away from the phenyl group while in all the others, it is bent back over the phenyl group. In fact, in UNUWIK there is an H⋯H contact of 2.4 Å between the phenyl ring of the benzyl group and that attached to the imidazole ring.

## Synthesis and crystallization   

To a solution of 4-(6-bromo-3*H*-imidazo[4,5-b]pyridin-2-yl)-*N*,*N*-di­methyl­aniline (0.4 g, 1.25 mmol), 2.2 equivalents of potassium carbonate (0.38 g, 2.75 mmol) and 0.2 equivalents of tetra-*n*-butyl ammonium bromide (BTBA) (0.061 g, 0.187 mmol) in 40 ml of DMF were added in small portions to 1.5 equivalent of the 1,6-di­bromo­dodeca­nedihalogenated reagent, and the mixture was stirred magnetically at room temperature for 48 h. After removal of the salts and evaporation of DMF under reduced pressure, the product was subjected to separation by chromatography on a column of silica gel using a mixture of hexa­ne/di­chloro­methane = 1/4 (*v*/*v*) as the mobile phase. Brown single crystals suitable for X-ray diffraction were obtained by evaporation of a di­chloro­methane/hexane solution (1:4 *v*/*v*).

## Refinement   

Crystal data, data collection and structure refinement details are summarized in Table 4 ▸. Hydrogen atoms were included as riding contributions in idealized positions (C—H = 0.95–0.99 Å) with *U*_iso_(H) = 1.2*U*_eq_(C) or 1.5*U*_eq_(C-meth­yl).

## Supplementary Material

Crystal structure: contains datablock(s) I, global. DOI: 10.1107/S2056989020008889/wm5570sup1.cif

Structure factors: contains datablock(s) I. DOI: 10.1107/S2056989020008889/wm5570Isup2.hkl

Supporting information file. DOI: 10.1107/S2056989020008889/wm5570Isup3.cdx

Supporting information file. DOI: 10.1107/S2056989020008889/wm5570Isup4.cml

CCDC reference: 2013416

Additional supporting information:  crystallographic information; 3D view; checkCIF report

## Figures and Tables

**Table 1:** Hydrogen-bond geometry (Å, °) *Cg*3 is the centroid of the C7–C12 phenyl ring.

*D*—H⋯*A*	*D*—H	H⋯*A*	*D*⋯*A*	*D*—H⋯*A*
C14—H14*B*⋯*Cg*3^vi^	0.99	2.60	3.502 (4)	151
C15—H15*A*⋯*Cg*3^ii^	0.99	2.94	3.904 (4)	165

Symmetry codes: (ii) 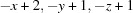; (vi) 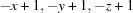.

**Table 2:** Comparison of selected (X-ray and DFT) bond length and angles (Å, °)

	X-ray	B3LYP/6–311G(d,p)
Br1—C3	1.905 (3)	1.95402
Br2—C18	1.993 (4)	2.02785
N1—C5	1.346 (4)	1.33429
N1—C4	1.347 (4)	1.37682
N2—C5	1.373 (4)	1.39261
N2—C6	1.387 (4)	1.39443
N2—C13	1.497 (4)	1.47275
N3—C6	1.321 (4)	1.33277
N3—C1	1.381 (4)	1.38362
N4—C10	1.373 (4)	1.39418
N4—C20	1.449 (5)	1.46048
N4—C19	1.464 (5)	1.46022
C5—N1—C4	112.1 (3)	114.50142
C5—N2—C6	105.4 (2)	108.33854
C5—N2—C13	124.1 (2)	118.46297
C6—N2—C13	129.9 (3)	128.08505
C6—N3—C1	105.2 (2)	107.12596
C10—N4—C20	119.7 (3)	120.14593
C10—N4—C19	120.1 (3)	120.10245
C20—N4—C19	117.7 (3)	119.74504
N3—C1—C2	131.6 (3)	134.19166
N3—C1—C5	109.6 (3)	106.29375

**Table 3:** Calculated energies

Mol­ecular Energy	Compound (I)
Total Energy *TE* (eV)	−167186.456
*E*_HOMO_ (eV)	−3.1033
*E*_LUMO_ (eV)	−0.7442
Gap, *ΔE* (eV)	2.3591
Dipole moment, *μ* (Debye)	6.1953
Ionization potential, *I* (eV)	3.1033
Electron affinity, *A*	0.7442
Electronegativity, *χ*	1.9237
Hardness, *η*	1.1796
Electrophilicity, index *ω*	1.5687
Softness, *σ*	0.8478
Fraction of electron transferred, *ΔN*	2.1518

**Table 4:** Experimental details

Crystal data	
Chemical formula	C_20_H_24_Br_2_N_4_
*M*_r_	480.25
Crystal system, space group	Triclinic, *P* 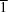
Temperature (K)	150
*a*, *b*, *c* (Å)	8.1488 (11), 11.2243 (15), 12.6378 (17)
α, β, γ (°)	64.049 (2), 74.184 (2), 86.067 (2)
*V* (Å^3^)	998.3 (2)
*Z*	2
Radiation type	Mo *K*α
μ (mm^−1^)	4.07
Crystal size (mm)	0.35 × 0.21 × 0.20

Data collection	
Diffractometer	Bruker SMART APEX CCD
Absorption correction	Multi-scan (*SADABS*; Krause *et al.*, 2015 ▸)
*T*_min_, *T*_max_	0.41, 0.50
No. of measured, independent and observed [*I* > 2σ(*I*)] reflections	19397, 5325, 4168
*R*_int_	0.025
(sin θ/λ)_max_ (Å^−1^)	0.687

Refinement	
*R*[*F* ^2^ > 2σ(*F* ^2^)], *wR*(*F* ^2^), *S*	0.046, 0.140, 1.12
No. of reflections	5325
No. of parameters	237
H-atom treatment	H-atom parameters constrained
Δρ_max_, Δρ_min_ (e Å^−3^)	2.18, −0.81

Computer programs: *APEX3* and *SAINT* (Bruker, 2016 ▸), *SHELXT* (Sheldrick, 2015*a* ▸), *SHELXL2018/1* (Sheldrick, 2015*b* ▸), *DIAMOND* (Brandenburg & Putz, 2012 ▸) and *publCIF* (Westrip, 2010 ▸).

## supplementary crystallographic information

### Crystal data

**Table:**

C_20_H_24_Br_2_N_4_	*Z* = 2
*M**_r_* = 480.25	*F*(000) = 484
Triclinic, *P*1	*D*_x_ = 1.598 Mg m^−^^3^
*a* = 8.1488 (11) Å	Mo *K*α radiation, λ = 0.71073 Å
*b* = 11.2243 (15) Å	Cell parameters from 9203 reflections
*c* = 12.6378 (17) Å	θ = 2.6–29.1°
α = 64.049 (2)°	µ = 4.07 mm^−^^1^
β = 74.184 (2)°	*T* = 150 K
γ = 86.067 (2)°	Block, brown
*V* = 998.3 (2) Å^3^	0.35 × 0.21 × 0.20 mm

### Data collection

**Table:**

Bruker SMART APEX CCD diffractometer	5325 independent reflections
Radiation source: fine-focus sealed tube	4168 reflections with *I* > 2σ(*I*)
Graphite monochromator	*R*_int_ = 0.025
Detector resolution: 8.3333 pixels mm^-1^	θ_max_ = 29.2°, θ_min_ = 1.9°
φ and ω scans	*h* = −11→11
Absorption correction: multi-scan (*SADABS*; Krause *et al.*, 2015)	*k* = −15→15
*T*_min_ = 0.41, *T*_max_ = 0.50	*l* = −17→17
19397 measured reflections	

### Refinement

**Table:**

Refinement on *F*^2^	Primary atom site location: dual
Least-squares matrix: full	Secondary atom site location: difference Fourier map
*R*[*F*^2^ > 2σ(*F*^2^)] = 0.046	Hydrogen site location: inferred from neighbouring sites
*wR*(*F*^2^) = 0.140	H-atom parameters constrained
*S* = 1.12	*w* = 1/[σ^2^(*F*_o_^2^) + (0.0785*P*)^2^ + 0.5344*P*] where *P* = (*F*_o_^2^ + 2*F*_c_^2^)/3
5325 reflections	(Δ/σ)_max_ < 0.001
237 parameters	Δρ_max_ = 2.18 e Å^−^^3^
0 restraints	Δρ_min_ = −0.80 e Å^−^^3^

### Special details

**Table:**

Experimental. The diffraction data were obtained from 3 sets of 400 frames, each of width 0.5° in ω, collected at φ = 0.00, 90.00 and 180.00° and 2 sets of 800 frames, each of width 0.45° in φ, collected at ω = –30.00 and 210.00°. The scan time was 20 sec/frame.
Geometry. All esds (except the esd in the dihedral angle between two l.s. planes) are estimated using the full covariance matrix. The cell esds are taken into account individually in the estimation of esds in distances, angles and torsion angles; correlations between esds in cell parameters are only used when they are defined by crystal symmetry. An approximate (isotropic) treatment of cell esds is used for estimating esds involving l.s. planes.
Refinement. Refinement of F^2^ against ALL reflections. The weighted R-factor wR and goodness of fit S are based on F^2^, conventional R-factors R are based on F, with F set to zero for negative F^2^. The threshold expression of F^2^ > 2sigma(F^2^) is used only for calculating R-factors(gt) etc. and is not relevant to the choice of reflections for refinement. R-factors based on F^2^ are statistically about twice as large as those based on F, and R- factors based on ALL data will be even larger. H-atoms attached to carbon were placed in calculated positions (C—H = 0.95 - 0.99 Å). All were included as riding contributions with isotropic displacement parameters 1.2 - 1.5 times those of the attached atoms.

### Fractional atomic coordinates and isotropic or equivalent isotropic displacement parameters (Å^2^)

**Table:**

	*x*	*y*	*z*	*U*_iso_*/*U*_eq_	
Br1	0.38201 (6)	−0.28330 (4)	0.92693 (3)	0.05798 (15)	
Br2	0.95197 (4)	0.93260 (3)	0.34919 (3)	0.04034 (12)	
N1	0.6232 (4)	0.0771 (3)	0.8277 (2)	0.0434 (7)	
N2	0.6823 (4)	0.2652 (3)	0.6288 (2)	0.0367 (6)	
N3	0.5754 (3)	0.1802 (3)	0.5275 (2)	0.0348 (6)	
N4	0.8327 (4)	0.7359 (3)	0.0600 (3)	0.0487 (7)	
C1	0.5486 (4)	0.0877 (3)	0.6478 (3)	0.0305 (6)	
C2	0.4742 (4)	−0.0402 (3)	0.7100 (3)	0.0375 (7)	
H2	0.426342	−0.080259	0.671849	0.045*	
C3	0.4747 (4)	−0.1051 (3)	0.8308 (3)	0.0382 (7)	
C4	0.5490 (5)	−0.0466 (3)	0.8856 (3)	0.0457 (8)	
H4	0.547009	−0.097433	0.969032	0.055*	
C5	0.6152 (4)	0.1393 (3)	0.7109 (3)	0.0348 (6)	
C6	0.6541 (4)	0.2842 (3)	0.5191 (3)	0.0287 (6)	
C7	0.7070 (4)	0.4037 (3)	0.4039 (3)	0.0298 (6)	
C8	0.7208 (4)	0.5323 (3)	0.3942 (3)	0.0356 (6)	
H8	0.698994	0.544398	0.466483	0.043*	
C9	0.7655 (4)	0.6417 (3)	0.2817 (3)	0.0391 (7)	
H9	0.775946	0.727231	0.278106	0.047*	
C10	0.7958 (4)	0.6281 (3)	0.1725 (3)	0.0355 (6)	
C11	0.7797 (4)	0.4999 (3)	0.1826 (3)	0.0371 (7)	
H11	0.799081	0.487401	0.110577	0.044*	
C12	0.7362 (4)	0.3916 (3)	0.2950 (3)	0.0357 (7)	
H12	0.725654	0.306081	0.298581	0.043*	
C13	0.7850 (4)	0.3494 (3)	0.6551 (3)	0.0370 (7)	
H13A	0.855360	0.417953	0.577241	0.044*	
H13B	0.863039	0.293447	0.702436	0.044*	
C14	0.6702 (5)	0.4158 (3)	0.7268 (3)	0.0401 (7)	
H14A	0.621256	0.348586	0.811797	0.048*	
H14B	0.574420	0.452669	0.690150	0.048*	
C15	0.7645 (5)	0.5269 (3)	0.7290 (3)	0.0410 (7)	
H15A	0.879431	0.499333	0.739215	0.049*	
H15B	0.701343	0.543649	0.799681	0.049*	
C16	0.7824 (4)	0.6547 (3)	0.6111 (3)	0.0374 (7)	
H16A	0.865311	0.642510	0.543472	0.045*	
H16B	0.670741	0.670141	0.591780	0.045*	
C17	0.8412 (5)	0.7779 (3)	0.6165 (3)	0.0386 (7)	
H17A	0.779730	0.776918	0.696102	0.046*	
H17B	0.964794	0.775162	0.610989	0.046*	
C18	0.8093 (5)	0.9053 (3)	0.5144 (3)	0.0406 (7)	
H18A	0.832848	0.981153	0.529282	0.049*	
H18B	0.687247	0.904205	0.516041	0.049*	
C19	0.8714 (6)	0.7165 (4)	−0.0514 (3)	0.0552 (10)	
H19A	0.972594	0.664450	−0.055484	0.083*	
H19B	0.893616	0.802985	−0.123010	0.083*	
H19C	0.773928	0.668979	−0.050252	0.083*	
C20	0.8846 (7)	0.8629 (4)	0.0483 (4)	0.0683 (13)	
H20A	0.793094	0.891955	0.098904	0.103*	
H20B	0.907455	0.928588	−0.037358	0.103*	
H20C	0.988418	0.854195	0.075477	0.103*	

### Atomic displacement parameters (Å^2^)

**Table:**

	*U*^11^	*U*^22^	*U*^33^	*U*^12^	*U*^13^	*U*^23^
Br1	0.0870 (3)	0.0396 (2)	0.0379 (2)	−0.02465 (19)	−0.01769 (19)	−0.00405 (15)
Br2	0.0480 (2)	0.03483 (19)	0.03595 (19)	−0.00702 (14)	−0.00713 (14)	−0.01459 (14)
N1	0.070 (2)	0.0335 (14)	0.0294 (13)	−0.0024 (13)	−0.0171 (13)	−0.0132 (11)
N2	0.0564 (17)	0.0280 (13)	0.0286 (12)	−0.0006 (11)	−0.0137 (11)	−0.0133 (10)
N3	0.0453 (15)	0.0297 (13)	0.0298 (12)	−0.0058 (11)	−0.0160 (11)	−0.0087 (10)
N4	0.068 (2)	0.0363 (15)	0.0358 (15)	−0.0161 (14)	−0.0163 (14)	−0.0067 (12)
C1	0.0333 (14)	0.0294 (14)	0.0289 (14)	0.0005 (11)	−0.0102 (11)	−0.0118 (11)
C2	0.0408 (17)	0.0364 (16)	0.0351 (16)	−0.0080 (13)	−0.0134 (13)	−0.0122 (13)
C3	0.0507 (19)	0.0274 (14)	0.0321 (15)	−0.0053 (13)	−0.0081 (13)	−0.0096 (12)
C4	0.074 (2)	0.0338 (17)	0.0284 (15)	−0.0023 (16)	−0.0110 (15)	−0.0141 (13)
C5	0.0517 (18)	0.0257 (14)	0.0290 (14)	0.0020 (13)	−0.0107 (13)	−0.0137 (11)
C6	0.0329 (14)	0.0303 (14)	0.0266 (13)	0.0023 (11)	−0.0100 (11)	−0.0144 (11)
C7	0.0328 (14)	0.0272 (14)	0.0293 (14)	0.0023 (11)	−0.0127 (11)	−0.0099 (11)
C8	0.0484 (18)	0.0303 (15)	0.0341 (15)	−0.0027 (13)	−0.0182 (13)	−0.0147 (12)
C9	0.056 (2)	0.0262 (14)	0.0380 (16)	−0.0069 (13)	−0.0218 (15)	−0.0100 (12)
C10	0.0364 (16)	0.0331 (15)	0.0337 (15)	−0.0062 (12)	−0.0127 (12)	−0.0087 (12)
C11	0.0449 (18)	0.0364 (16)	0.0317 (15)	0.0008 (13)	−0.0106 (13)	−0.0161 (13)
C12	0.0485 (18)	0.0290 (15)	0.0337 (16)	0.0006 (13)	−0.0121 (13)	−0.0166 (13)
C13	0.0416 (17)	0.0328 (15)	0.0387 (16)	0.0016 (13)	−0.0165 (13)	−0.0140 (13)
C14	0.0522 (19)	0.0370 (17)	0.0324 (16)	−0.0025 (14)	−0.0114 (14)	−0.0157 (13)
C15	0.060 (2)	0.0330 (16)	0.0379 (17)	−0.0038 (14)	−0.0218 (15)	−0.0167 (13)
C16	0.0467 (18)	0.0334 (16)	0.0367 (16)	−0.0051 (13)	−0.0157 (13)	−0.0157 (13)
C17	0.0475 (18)	0.0349 (16)	0.0422 (18)	0.0015 (14)	−0.0184 (14)	−0.0210 (14)
C18	0.0502 (19)	0.0333 (16)	0.0441 (18)	0.0022 (14)	−0.0165 (15)	−0.0199 (14)
C19	0.071 (3)	0.049 (2)	0.0316 (17)	−0.0067 (19)	−0.0104 (17)	−0.0059 (15)
C20	0.112 (4)	0.0352 (19)	0.051 (2)	−0.026 (2)	−0.031 (2)	−0.0034 (17)

### Geometric parameters (Å, º)

**Table:**

Br1—C3	1.905 (3)	C11—C12	1.376 (4)
Br2—C18	1.993 (4)	C11—H11	0.9500
N1—C5	1.346 (4)	C12—H12	0.9500
N1—C4	1.347 (4)	C13—C14	1.512 (5)
N2—C5	1.373 (4)	C13—H13A	0.9900
N2—C6	1.387 (4)	C13—H13B	0.9900
N2—C13	1.497 (4)	C14—C15	1.523 (4)
N3—C6	1.321 (4)	C14—H14A	0.9900
N3—C1	1.381 (4)	C14—H14B	0.9900
N4—C10	1.373 (4)	C15—C16	1.530 (5)
N4—C20	1.449 (5)	C15—H15A	0.9900
N4—C19	1.464 (5)	C15—H15B	0.9900
C1—C2	1.385 (4)	C16—C17	1.528 (4)
C1—C5	1.396 (4)	C16—H16A	0.9900
C2—C3	1.376 (4)	C16—H16B	0.9900
C2—H2	0.9500	C17—C18	1.514 (5)
C3—C4	1.399 (5)	C17—H17A	0.9900
C4—H4	0.9500	C17—H17B	0.9900
C6—C7	1.461 (4)	C18—H18A	0.9900
C7—C12	1.396 (4)	C18—H18B	0.9900
C7—C8	1.404 (4)	C19—H19A	0.9800
C8—C9	1.383 (4)	C19—H19B	0.9800
C8—H8	0.9500	C19—H19C	0.9800
C9—C10	1.408 (4)	C20—H20A	0.9800
C9—H9	0.9500	C20—H20B	0.9800
C10—C11	1.401 (4)	C20—H20C	0.9800
			
Br1···C11^i^	3.734 (4)	C9···H20A	2.72
Br2···N1^ii^	3.597 (3)	C9···H14B^vi^	2.91
Br2···N2^ii^	3.591 (3)	C9···H20C	2.89
Br2···C5^ii^	3.515 (4)	C11···H19C	2.73
Br2···C13^ii^	3.711 (4)	C11···H19A	2.81
Br1···H14A^iii^	3.05	C12···H14B^vi^	2.99
Br1···H11^i^	3.07	C13···H8	2.66
Br2···H16A	3.08	C14···H8	2.90
Br2···H13B^ii^	3.09	C16···H8	2.85
Br2···H18A^iv^	3.06	C16···H13A	2.87
Br2···H19B^v^	3.11	C19···H11	2.47
N1···C4^iii^	3.380 (5)	C20···H9	2.54
N1···H13B	2.80	C20···H20B^v^	2.92
N1···H4^iii^	2.67	C20···H20C^v^	2.97
N2···H8	2.89	H2···H18B^vii^	2.53
N3···H12	2.57	H8···H13A	2.14
N3···H16B^vi^	2.84	H8···H14B	2.47
N3···H18B^vi^	2.68	H8···H16A	2.42
C1···C18^vii^	3.467 (5)	H8···H16B	2.50
C2···C18^vii^	3.370 (5)	H9···H20A	2.18
C4···C19^viii^	3.597 (6)	H9···H20C	2.51
C4···C4^iii^	3.372 (5)	H11···C19	2.47
C8···C13	3.198 (5)	H11···H19C	2.21
C20···C20^v^	3.279 (7)	H13A···H16A	2.37
C1···H18A^vii^	2.89	H13B···H15A	2.57
C2···H18B^vii^	2.88	H14B···H16B	2.28
C4···H4^iii^	2.87	H15B···H17A	2.40
C7···H14B^vi^	2.93	H16B···H18B	2.37
C7···H13A	2.84	H19B···H20B	2.14
C8···H13A	2.61	H20B···H20C^v^	2.43
C8···H14B^vi^	2.85		
			
C5—N1—C4	112.1 (3)	C14—C13—H13A	109.4
C5—N2—C6	105.4 (2)	N2—C13—H13B	109.4
C5—N2—C13	124.1 (2)	C14—C13—H13B	109.4
C6—N2—C13	129.9 (3)	H13A—C13—H13B	108.0
C6—N3—C1	105.2 (2)	C13—C14—C15	112.5 (3)
C10—N4—C20	119.7 (3)	C13—C14—H14A	109.1
C10—N4—C19	120.1 (3)	C15—C14—H14A	109.1
C20—N4—C19	117.7 (3)	C13—C14—H14B	109.1
N3—C1—C2	131.6 (3)	C15—C14—H14B	109.1
N3—C1—C5	109.6 (3)	H14A—C14—H14B	107.8
C2—C1—C5	118.7 (3)	C14—C15—C16	111.1 (3)
C3—C2—C1	115.2 (3)	C14—C15—H15A	109.4
C3—C2—H2	122.4	C16—C15—H15A	109.4
C1—C2—H2	122.4	C14—C15—H15B	109.4
C2—C3—C4	122.1 (3)	C16—C15—H15B	109.4
C2—C3—Br1	120.0 (2)	H15A—C15—H15B	108.0
C4—C3—Br1	117.9 (2)	C17—C16—C15	114.3 (3)
N1—C4—C3	124.2 (3)	C17—C16—H16A	108.7
N1—C4—H4	117.9	C15—C16—H16A	108.7
C3—C4—H4	117.9	C17—C16—H16B	108.7
N1—C5—N2	125.4 (3)	C15—C16—H16B	108.7
N1—C5—C1	127.6 (3)	H16A—C16—H16B	107.6
N2—C5—C1	106.8 (3)	C18—C17—C16	112.2 (3)
N3—C6—N2	113.0 (3)	C18—C17—H17A	109.2
N3—C6—C7	121.8 (2)	C16—C17—H17A	109.2
N2—C6—C7	125.3 (3)	C18—C17—H17B	109.2
C12—C7—C8	117.0 (3)	C16—C17—H17B	109.2
C12—C7—C6	118.3 (3)	H17A—C17—H17B	107.9
C8—C7—C6	124.6 (3)	C17—C18—Br2	113.4 (2)
C9—C8—C7	121.5 (3)	C17—C18—H18A	108.9
C9—C8—H8	119.3	Br2—C18—H18A	108.9
C7—C8—H8	119.3	C17—C18—H18B	108.9
C8—C9—C10	121.0 (3)	Br2—C18—H18B	108.9
C8—C9—H9	119.5	H18A—C18—H18B	107.7
C10—C9—H9	119.5	N4—C19—H19A	109.5
N4—C10—C11	120.6 (3)	N4—C19—H19B	109.5
N4—C10—C9	122.0 (3)	H19A—C19—H19B	109.5
C11—C10—C9	117.3 (3)	N4—C19—H19C	109.5
C12—C11—C10	121.2 (3)	H19A—C19—H19C	109.5
C12—C11—H11	119.4	H19B—C19—H19C	109.5
C10—C11—H11	119.4	N4—C20—H20A	109.5
C11—C12—C7	122.0 (3)	N4—C20—H20B	109.5
C11—C12—H12	119.0	H20A—C20—H20B	109.5
C7—C12—H12	119.0	N4—C20—H20C	109.5
N2—C13—C14	111.0 (3)	H20A—C20—H20C	109.5
N2—C13—H13A	109.4	H20B—C20—H20C	109.5
			
C6—N3—C1—C2	−179.0 (3)	N3—C6—C7—C12	−24.9 (4)
C6—N3—C1—C5	−0.6 (3)	N2—C6—C7—C12	153.7 (3)
N3—C1—C2—C3	178.0 (3)	N3—C6—C7—C8	151.0 (3)
C5—C1—C2—C3	−0.3 (4)	N2—C6—C7—C8	−30.4 (5)
C1—C2—C3—C4	−1.5 (5)	C12—C7—C8—C9	−1.6 (5)
C1—C2—C3—Br1	−178.4 (2)	C6—C7—C8—C9	−177.5 (3)
C5—N1—C4—C3	1.0 (5)	C7—C8—C9—C10	1.2 (5)
C2—C3—C4—N1	1.2 (6)	C20—N4—C10—C11	−166.9 (4)
Br1—C3—C4—N1	178.2 (3)	C19—N4—C10—C11	−5.7 (5)
C4—N1—C5—N2	−178.8 (3)	C20—N4—C10—C9	15.9 (6)
C4—N1—C5—C1	−3.1 (5)	C19—N4—C10—C9	177.1 (3)
C6—N2—C5—N1	176.1 (3)	C8—C9—C10—N4	177.0 (3)
C13—N2—C5—N1	4.1 (5)	C8—C9—C10—C11	−0.3 (5)
C6—N2—C5—C1	−0.3 (3)	N4—C10—C11—C12	−177.4 (3)
C13—N2—C5—C1	−172.3 (3)	C9—C10—C11—C12	−0.1 (5)
N3—C1—C5—N1	−175.8 (3)	C10—C11—C12—C7	−0.3 (5)
C2—C1—C5—N1	2.9 (5)	C8—C7—C12—C11	1.2 (5)
N3—C1—C5—N2	0.5 (4)	C6—C7—C12—C11	177.4 (3)
C2—C1—C5—N2	179.2 (3)	C5—N2—C13—C14	−80.8 (4)
C1—N3—C6—N2	0.4 (3)	C6—N2—C13—C14	109.3 (4)
C1—N3—C6—C7	179.1 (3)	N2—C13—C14—C15	−166.1 (3)
C5—N2—C6—N3	0.0 (3)	C13—C14—C15—C16	80.7 (4)
C13—N2—C6—N3	171.4 (3)	C14—C15—C16—C17	167.7 (3)
C5—N2—C6—C7	−178.8 (3)	C15—C16—C17—C18	−163.7 (3)
C13—N2—C6—C7	−7.4 (5)	C16—C17—C18—Br2	−67.2 (3)

Symmetry codes: (i) −*x*+1, −*y*, −*z*+1; (ii) −*x*+2, −*y*+1, −*z*+1; (iii) −*x*+1, −*y*, −*z*+2; (iv) −*x*+2, −*y*+2, −*z*+1; (v) −*x*+2, −*y*+2, −*z*; (vi) −*x*+1, −*y*+1, −*z*+1; (vii) *x*, *y*−1, *z*; (viii) *x*, *y*−1, *z*+1.

### Hydrogen-bond geometry (Å, º)

*Cg*3 is the centroid of the C7–C12 phenyl ring.

**Table:**

*D*—H···*A*	*D*—H	H···*A*	*D*···*A*	*D*—H···*A*
C14—H14*B*···*Cg*3^vi^	0.99	2.60	3.502 (4)	151
C15—H15*A*···*Cg*3^ii^	0.99	2.94	3.904 (4)	165

Symmetry codes: (ii) −*x*+2, −*y*+1, −*z*+1; (vi) −*x*+1, −*y*+1, −*z*+1.
